# The diverse effects of side reactions on the persistence of autocatalytic cycles

**DOI:** 10.1098/rsif.2026.0308

**Published:** 2026-10-14

**Authors:** Zhen Peng, Emily Spencer, David Baum

**Affiliations:** ^1^ State Key Laboratory of Bioreactor Engineering, School of Biotechnology, East China University of Science and Technology, Shanghai, People's Republic of China; ^2^ Wisconsin Institute for Discovery, University of Wisconsin–Madison, Madison, WI, USA; ^3^ Department of Biology, University of Wisconsin–Madison, Madison, WI, USA

**Keywords:** autocatalysis, origins of life, metabolism-first theories, chemical reaction networks, stochastic chemical kinetics

## Abstract

A key criticism of metabolism-first theories of abiogenesis is that, in the absence of specialized catalysts, side reactions would convert too many autocatalysts to inert by-products for autocatalytic systems to persist. Here, we use algebraic analysis and numerical simulations to explore the effects of side reactions on the persistence of simple autocatalytic systems. For an autocatalytic reaction coupled to a side reaction, whenever a component reaction is strictly irreversible, which is technically impossible but frequently considered an acceptable approximation, side reactions harm autocatalytic systems. But if all reactions are assumed reversible, perhaps with unequal forward and reverse rate constants, side reactions can have a wide range of effects—they can be harmful, neutral, beneficial, or harmful in the short term but beneficial in the longer term. These effects arise in the presence of reversible reactions, because side products can also serve as a reservoir from which autocatalysts can be re-derived. We then use algebraic analysis to confirm that such diverse effects also apply to a larger autocatalytic cycle coupled to more side reactions. Our results show that the effects of side reactions on autocatalytic systems do not provide sufficient grounds to reject metabolism-first models.

## 1. Introduction

Metabolism-first theories of the origins of life posit that self-sustaining reaction networks pre-dated the appearance of catalytic polymers [1–8]. These theories have been countered by two major criticisms. First, to be self-sustaining, a reaction network must be based on autocatalytic cycles that continuously convert environmental nutrients into more members of the cycles. Without specialized catalysts, it is argued that side reactions leading from autocatalytic cycles to inert by-products would extract too many materials for the cycles to persist [9–11]. Second, in the absence of genetic encoding, prebiotic metabolic networks would have no, or very limited, ability to adaptively evolve [6,7,9,10]. The validity of these two criticisms is of great importance for understanding the origins of life. In particular, if the first criticism holds, then we are forced to conclude that life can only originate in settings where catalytically discriminating polymers arise spontaneously, greatly narrowing the plausible conditions for abiogenesis.

It has been shown that, even if side reactions irreversibly produce inert by-products, they need not completely disrupt autocatalysis as long as the food molecules (i.e. species that are consumed during autocatalysis) are maintained at a sufficiently high concentration [12]. Additionally, the assumption that side reactions necessarily lead to inert by-products has been questioned. For example, it has been proposed that side reactions might lead to the synthesis of catalysts, much as intermediates in the autocatalytic reductive tricarboxylic acid cycle are precursors of amino acids [13], which are the building blocks of peptide catalysts [14]. Shapiro [14] also suggested that side reactions might form alternative paths between nodes within an autocatalytic cycle, in which case they would not prevent autocatalysis but redirect flux through different member species. In addition, studies based on the reaction–diffusion model and its variants showed that spatial structures may attenuate the harmful effects of molecular parasites and even turn parasites into beneficial factors [15–18]. For example, Froese, Ikegami, and Virgo [19–21] considered third-order autocatalytic reactions using reaction–diffusion models and showed that an autocatalytic reaction with a parasitic, autocatalytic side reaction may together form a motile system that better forages for environmental resources than the host cycle alone.

In contrast with previous studies, which considered additional factors that can protect autocatalytic cycles from the negative effects of side reactions, we set out to identify the general principles determining the influence of side reactions on the persistence of autocatalytic cycles, once activated. To do this, we thoroughly analyse a simple system consisting of a single autocatalytic reaction with or without a side reaction. We considered bulk chemical kinetics without spatial structure, because most side-reaction-based criticisms of metabolism-first theories are framed in this context. Our analysis is based on two assumptions: (i) reactions are reversible, which is supported by the principle of microscopic reversibility [22–25], and (ii) reactions occur in open reactors, which is supported by the fact that life originated in open environments. On the basis of these realistic assumptions, the results of our analysis will be of general significance.

Our results not only confirmed that side reactions may have negative effects on autocatalysts, but also showed that, in open reactors with reversible reactions, side reactions are not always lethal and, in some circumstances, can even help the autocatalytic system persist. In cases where the expected steady-state concentrations are too low for mass-action kinetics to apply, we found cases where a reversible side reaction may be harmful soon after a new autocatalytic cycle has been seeded, but beneficial once it has been established. Using algebraic analysis, we confirmed that the general principles also apply to larger autocatalytic cycles coupled to more side reactions. Our results show that the effects of a side reaction on an autocatalytic system are complex and do not provide sufficient grounds for rejecting metabolism-first models.

## 2. Results

### 2.1. The model

In §2.2, we explored a combination of the simplest autocatalytic reaction A + F → 2A, or in the reversible case A + F ⇌ 2A, and the simplest side reaction A → B, or in the reversible case A ⇌ B. To more clearly evaluate the effect of the side reaction, we compared the A–B reaction system with the case of a simple autocatalytic reaction without a side reaction, C + F → 2C, or in the reversible case C + F ⇌ 2C. We modelled the reactions in an abstract continuous stirred tank reactor (CSTR) receiving a constant inflow of a food solution and concomitant outflow of all chemical species (figure 1).

**Figure 1.**
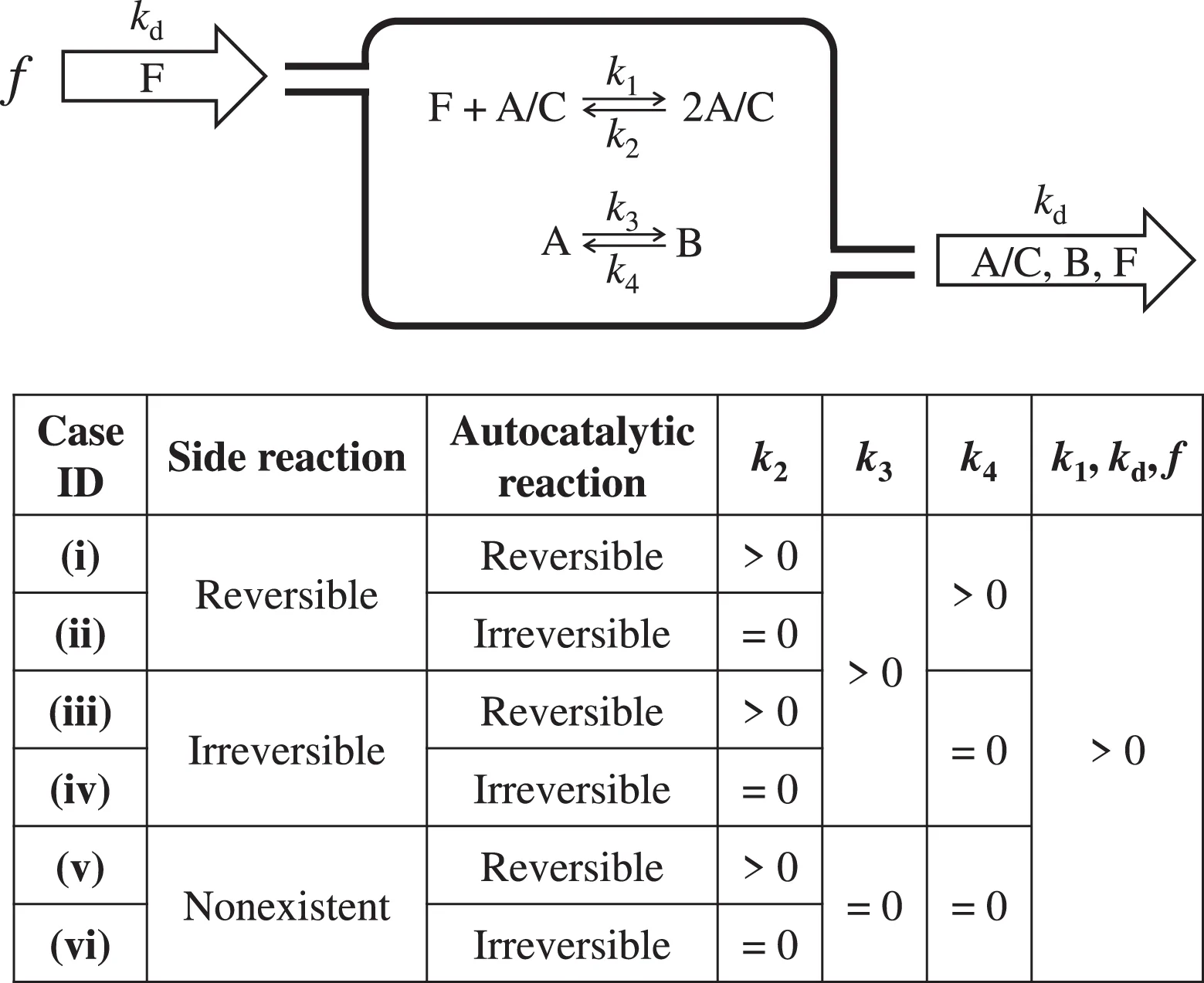
The relationship between model properties and parameter values. *f* is the concentration of food, F, in the external food source; *k*_d_ is the dilution rate of the reactor; *k*_1_ and *k*_2_ are reaction rate constants for the reversible autocatalytic reactions, A + F ⇌ 2A or C + F ⇌ 2C, whereas *k*_3_ and *k*_4_ are the rate constants for the reversible side reaction, A ⇌ B.

In situations where integer counts instead of moles are better measures of molecular quantities, stochastic kinetics provides a better approximation of real chemical processes than mass-action kinetics [26–29]. Consideration of stochastic kinetics is important for origins-of-life research because many origins-of-life hypotheses involve small compartments (e.g. porous rocks, coacervates) in which the number of molecules could be limited. Moreover, it has been noted that early autocatalytic processes would probably be triggered by a single or a few autocatalytic ‘seed’ molecules [30,31]. In such cases, autocatalyst viability requires that at least one molecule remains present, which is best explored using stochastic simulations. We used this approach in §2.3.

Finally, we used algebraic analysis to investigate a more complex reaction system consisting of a two-reaction autocatalytic cycle and three side reactions in a CSTR. This analysis was used to assess whether the general principles extracted from the simple combination of one autocatalytic reaction and one side reaction apply to more complex systems (§2.4).

### 2.2. Mass-action kinetics of a simple autocatalytic system

We first use mass-action kinetics to explore a reaction system consisting of an autocatalytic reaction and a side reaction. Denoting the steady-state concentrations of A, B and F by [A]_s_, [B]_s_ and [F]_s_, respectively, the steady-state concentrations can be shown to be either (supplementary material, appendix S2)

(2.1) $$\left\{ \begin{array}{l} {\left[A\right]}_{s}=0\\ {\left[B\right]}_{s}=0\\ {\left[F\right]}_{s}=f \end{array}\right.$$

or

(2.2) $$\left\{ \begin{array}{l} \;{\left[A\right]}_{s}=\frac{k_{1}k_{4}f+k_{d}k_{1}f-k_{d}k_{3}-k_{d}k_{4}-k_{d}^{2}}{k_{1}k_{3}+k_{1}k_{4}+k_{2}k_{4}+k_{d}k_{1}+k_{d}k_{2}}\;\\ \;{\left[B\right]}_{s}=\frac{k_{3}}{k_{d}+k_{4}}\;{\left[A\right]}_{s}\\ \;{\left[F\right]}_{s}=f-\frac{k_{d}+k_{3}+k_{4}}{k_{d}+k_{4}}{\left[A\right]}_{s} \end{array}\right.$$

Equations (2.1) and (2.2) correspond to the extinction and survival of the autocatalytic system, respectively.

Analysing the cases where the autocatalytic reaction and the side reaction are both reversible could be a little complicated, so let us start with simpler cases. Consider the cases where the side reaction is strictly irreversible (figure 1, cases (iii) and (iv), *k*_4_ = 0), which results in

(2.3) $$\left\{ \begin{array}{l} {\left[A\right]}_{s}=\frac{k_{d}k_{1}f-k_{d}k_{3}-k_{d}^{2}}{k_{1}k_{3}+k_{d}k_{1}+k_{d}k_{2}}\\ {\left[B\right]}_{s}=\frac{k_{3}}{k_{d}}\;{\left[A\right]}_{s}\\ {\left[F\right]}_{s}=f-\frac{k_{d}+k_{3}}{k_{d}}{\left[A\right]}_{s}. \end{array}\right.$$

In order for the autocatalytic system to survive, [A]_s_ must be positive, which can be shown to hold when

(2.4) $$k_{1}>\frac{k_{3}+k_{d}}{f}.$$

It is worth noting that, according to equation (2.4), when the food concentration is high and the dilution rate is low, *k*_3_ can be very large without causing extinction. Moreover, because equation (2.4) does not involve *k*_2_, we can conclude that the system can persist even if the autocatalytic reaction is thermodynamically disfavoured (i.e. with *k*_2_ > *k*_1_). These results show that even in the presence of an irreversible reaction, a slow and thermodynamically disfavoured autocatalytic cycle can persist once activated in an open environment.

To determine whether the presence of an irreversible side reaction necessarily decreases the steady-state concentration of the autocatalyst, we need to consider a reaction system with the same rate constant(s) for the autocatalytic reaction but no side reaction (figure 1, cases (v) and (vi)). Assuming mass-action kinetics and denoting the steady-state concentration of C by [C]_s_, the steady-state concentrations are (supplementary material, appendix S1) either

(2.5) $$\left\{ \begin{array}{c} {\left[C\right]}_{s}=0\\ {\left[F\right]}_{s}=f \end{array}\right.$$

or

(2.6) $$\left\{ \begin{array}{c} \;{\;\;\left[C\right]}_{s}=\frac{k_{1}f-k_{d}}{k_{1}+k_{2}}\;\\ \;{\;\;\left[F\right]}_{s}=f-{\left[C\right]}_{s.} \end{array}\right.$$

Comparing [A]_s_ with [C]_s_ in equations (2.3) and (2.6), it can be proved that [A]_s_ – [C]_s_ is strictly negative whether the autocatalytic reaction is reversible (figure 1, cases (iii) and (v); supplementary material, appendix S3) or irreversible (figure 1, cases (iv) and (vi); supplementary material, appendix S3). This means that the presence of an irreversible side reaction always lowers the steady-state concentration of an autocatalyst relative to expectation in the counterfactual of no side reaction.

For the case where the autocatalytic reaction is strictly irreversible, and the side reaction is reversible (figure 1, case (ii), *k*_2_ = 0), based on equation (2.2), the survival of the autocatalytic system corresponds to (supplementary material, appendix S4):

(2.7) $$\left\{ \begin{array}{l} \;{\left[A\right]}_{s}=\frac{k_{1}k_{4}f+k_{d}k_{1}f-k_{d}k_{3}-k_{d}k_{4}-k_{d}^{2}}{k_{1}k_{3}+k_{1}k_{4}+k_{d}k_{1}}\;\\ \;{\left[B\right]}_{s}=\frac{k_{3}}{k_{d}+k_{4}}\;{\left[A\right]}_{s}\\ \;{\left[F\right]}_{s}=f-\frac{k_{d}+k_{3}+k_{4}}{k_{d}+k_{4}}{\left[A\right]}_{s.} \end{array}\right.$$

For evaluating the influence of the side reaction on the A–B system, we used [A]_s_ + [B]_s_ rather than [A]_s_ alone. Comparing [A]_s_ + [B]_s_ with [C]_s_ is reasonable despite the fact [A]_s_ + [B]_s_ may not be linearly correlated with extinction probability. First, even if all A molecules are removed, the reaction B → A can still reintroduce A into the CSTR, which means that completely shutting down the autocatalytic A–B system requires completely removing both A and B from the CSTR. In other words, B molecules can now be viewed as indirect autocatalysts. Second, the extinction of an autocatalytic system under mass-action kinetics is likely to result not from frequent random fluctuation but from infrequent disturbances that remove direct and indirect autocatalysts in bulk. This means that while *k*_3_ and *k*_4_ may influence how fast the A–B system recovers from a disturbance, what primarily determines whether an autocatalytic system can survive a disturbance is whether the total pre-disturbance amount of direct and indirect autocatalysts (i.e. [A]_s_ + [B]_s_, or [C]_s_) is larger than the intensity of the disturbance.

According to equations (2.6) and (2.7) (note that *k*_2_ = 0), we get (supplementary material, appendix S4)

(2.8) $$\begin{array}{r} {\left[A\right]}_{s}+{\left[B\right]}_{s}-\;{\left[C\right]}_{s}=\frac{-k_{d}k_{3}}{k_{d}k_{1}+k_{1}k_{4}}<0. \end{array}$$

Therefore, the side reaction always harms the autocatalytic system. This conclusion is perhaps less intuitive, but readily explained: a strictly irreversible autocatalytic reaction with no side reaction has maximal efficiency of converting food to autocatalysts, so that any side reaction can only reduce its efficiency.

Now let us analyse the case where the autocatalytic reaction and the side reaction are both reversible (figure 1, case (i)). This is more realistic than the prior conditions because thermodynamics (particularly the principle of microscopic reversibility) dictates that every real elementary reaction needs to be reversible [22–25]. The standard free energy change of a reaction is Δ_r_*G*^⊖^ = –*RT*ln*K*, where *R* is the gas constant, *T* is the temperature, and *K* is the equilibrium constant. Had there been a strictly irreversible reaction, the rate constant of the reverse reaction would be zero and thus *K* = ∞, Δ_r_*G*^⊖^ = –∞, which violates the laws of thermodynamics.

According to equations (2.1), (2.2), (2.5) and (2.6), and the property of side products being indirect autocatalysts, we can define a parameter *U* whose sign indicates whether the net effect of a side reaction is positive (*U* > 0) or negative (*U* < 0),

(2.9) $$\begin{array}{l} U\left(k_{1},k_{2},k_{3},k_{4},k_{d},f\right)=\\ max\left(0,\;\frac{k_{d}+k_{3}+k_{4}}{k_{d}+k_{4}}\cdot \frac{k_{1}k_{4}f+k_{d}k_{1}f-k_{d}k_{3}-k_{d}k_{4}-k_{d}^{2}}{k_{1}k_{3}+k_{1}k_{4}+k_{2}k_{4}+k_{d}k_{1}+k_{d}k_{2}}\right)\\ -max\left(0,\;\frac{k_{1}f-k_{d}}{k_{1}+k_{2}}\right). \end{array}$$

As illustrated in figure 2 and table S1 in the supplementary material, we can readily find examples in which the side reaction is beneficial (figure 2, *U* > 0; supplementary material, table S1, case (a)), or harmful (figure 2, *U* < 0; supplementary material, table S1, case (b)), or even lethal (figure 2, *U* < 0 and [A]_s_ + [B]_s_ = 0). Taken together, these results show that, because a reversible side reaction creates an additional reservoir of chemical species that can support autocatalysis by raising system efficiency, side reactions can sometimes provide a net benefit to the autocatalytic system. From now on, we will only consider the cases where every elementary reaction is reversible.

**Figure 2.**
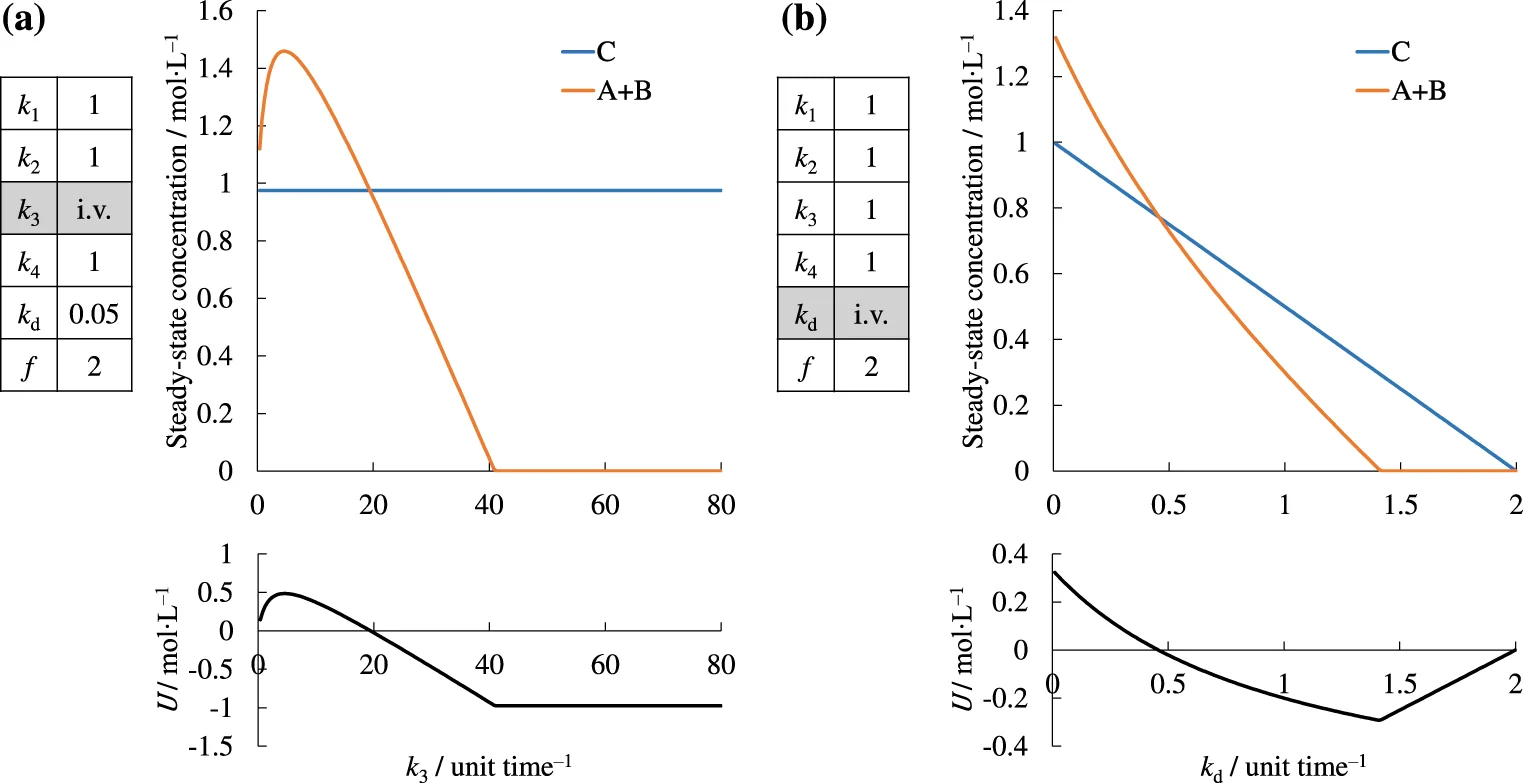
The influence of a side reaction on the persistence of a simple autocatalytic system varies with parameter values. Reaction settings are as described in figure 1, case (i) (reversible autocatalytic reaction and reversible side reaction) and case (v) (reversible autocatalytic reaction without side reaction). Two examples of the association between *U*(*k*_1_, *k*_2_, *k*_3_, *k*_4_, *k*_d_, *f*) and parameter values are shown. *k*'s: reaction rate constants; unit: mol^–1^ l unit time^–1^ for *k*_1_ and *k*_2_, unit time^–1^ for *k*_3_ and *k*_4_. *k*_d_: dilution rate; unit: unit time^–1^. *f*: food concentration of the external food source; unit: mol l^–1^. i.v., independent variable. (a) *k*_3_ serves as the independent variable. (b) *k*_d_ serves as the independent variable.

### 2.3. Stochastic chemical kinetics: Gillespie algorithm

As mentioned in §2.1, the Gillespie algorithm [26–29] is more appropriate than mass-action kinetics for studying the first emergence of prebiotic autocatalytic systems. By setting the volume of a CSTR to 1 l, we can convert expected concentrations of species ([A]_s_, [B]_s_ and [C]_s_) to expected molecule counts (*n*_s,A_, *n*_s,B_ and *n*_s,C_). As shown in supplementary material, table S1, cases (c) and (d), the expected steady-state counts of A, B and C molecules in reactors can be sufficiently low (i.e. well below 100) for mass-action kinetics to be unreliable. In both cases, *n*_s,A_ + *n*_s,B_ > *n*_s,C_, and thus one might intuitively predict that the side reaction is beneficial based on the experience with mass-action kinetics. To assess this intuition, we used numerical Gillespie simulations for 200 replicates each initialized with one A or C molecule and 500 F molecules. We then compared the survivorship of the A–B system (with side reaction) with the C system (without side reaction). Each replicate was run for 4000 time units (figure 3), and we tracked the number of replicates where the autocatalytic system went extinct (figure 3a and b) or survived (figure 3c and d).

**Figure 3.**
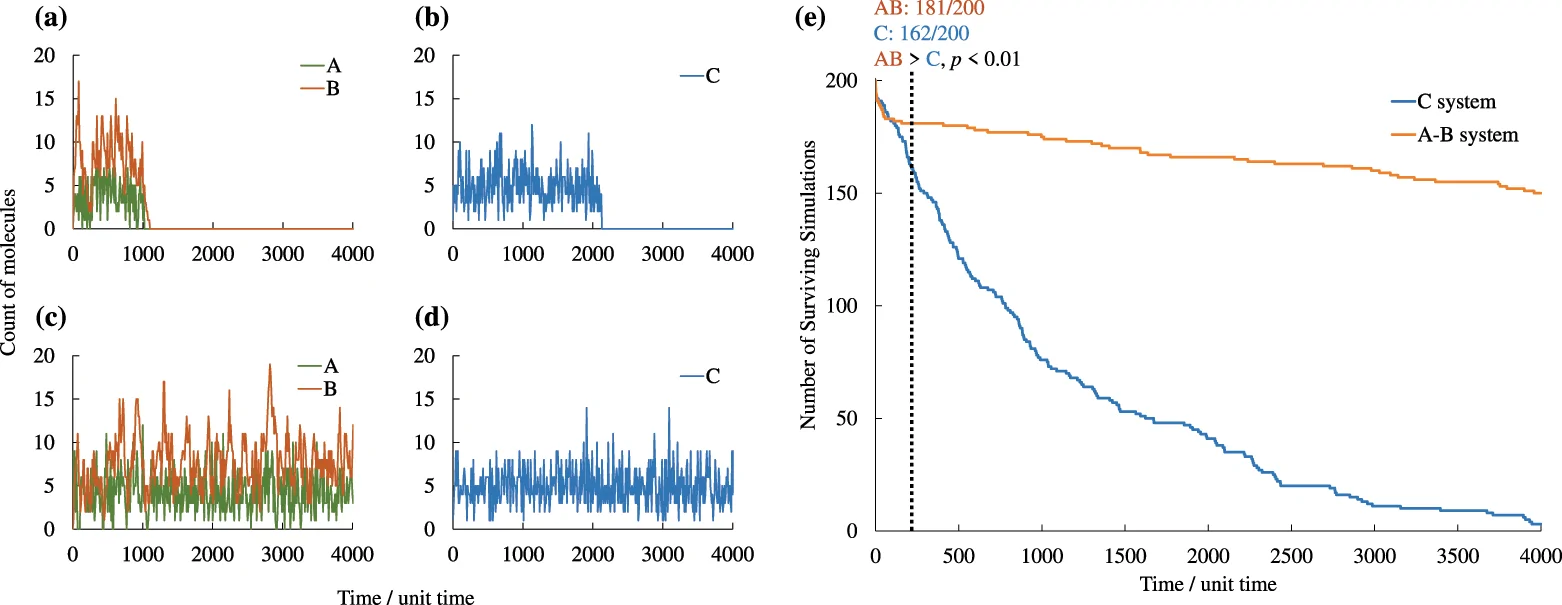
The autocatalysis-side-reaction system (A–B system) can be more persistent than the side-reaction-free system (C system). For each of the two reaction systems, 200 replicate simulations were performed. The parameters were set as specified in supplementary material, table S1, case (c); all simulations started with 500 F molecules and 1 A or C molecule. The expected steady-state count of A, B and C are 4.54, 8.16 and 4.83, respectively. (a) Example of an extinct A–B system. (b) Example of an extinct C system. (c) Example of a surviving A–B system. (d) Example of a surviving C system. For (a–d), the counts are shown every 10 time units. (e) The survivorship curves of the A–B and C systems, derived from the time points when extinction occurred.

For the parameters shown in supplementary material, table S1, case (c), the survivorship curves show that the A–B system survives better than the C system, meaning that the side reaction is indeed beneficial for the autocatalytic cycle (figure 3e). For the A–B system, 181 out of 200 replicates survive the first 211 time units, whereas for the C system, only 162 out of 200 replicates survive the first 211 time units. This is a significant difference based on Fisher's exact test (*p* < 0.01).

For the parameters shown in supplementary material, table S1, case (d), where *n*_s,A_ is considerably smaller than *n*_s,C_, the outcome is more complicated. As shown in figure 4, the side reaction significantly lowers the chance of the autocatalytic system establishing itself in the early part of the simulation. At the 1070th time unit, 128 of 200 replicates of the C system survive, which is significantly more than the 101 surviving replicates for the A–B system (Fisher's exact test, *p* < 0.01). However, as predicted on the basis of mass-action kinetics, the post-establishment survivorship of the A–B system is higher. At the 2678th time unit, all 101 of 200 replicates of the A–B system still survive, which is significantly more than the 74 surviving replicates of the C system (Fisher's exact test, *p* < 0.01). In other words, with the parameter values set as supplementary material, table S1, case (d), the autocatalytic system involving a side reaction has a higher probability of extinction when the system is first initiated but, once the system accumulates enough B molecules to evade immediate extinction, it then has a significantly higher probability of persisting in the long term.

**Figure 4.**
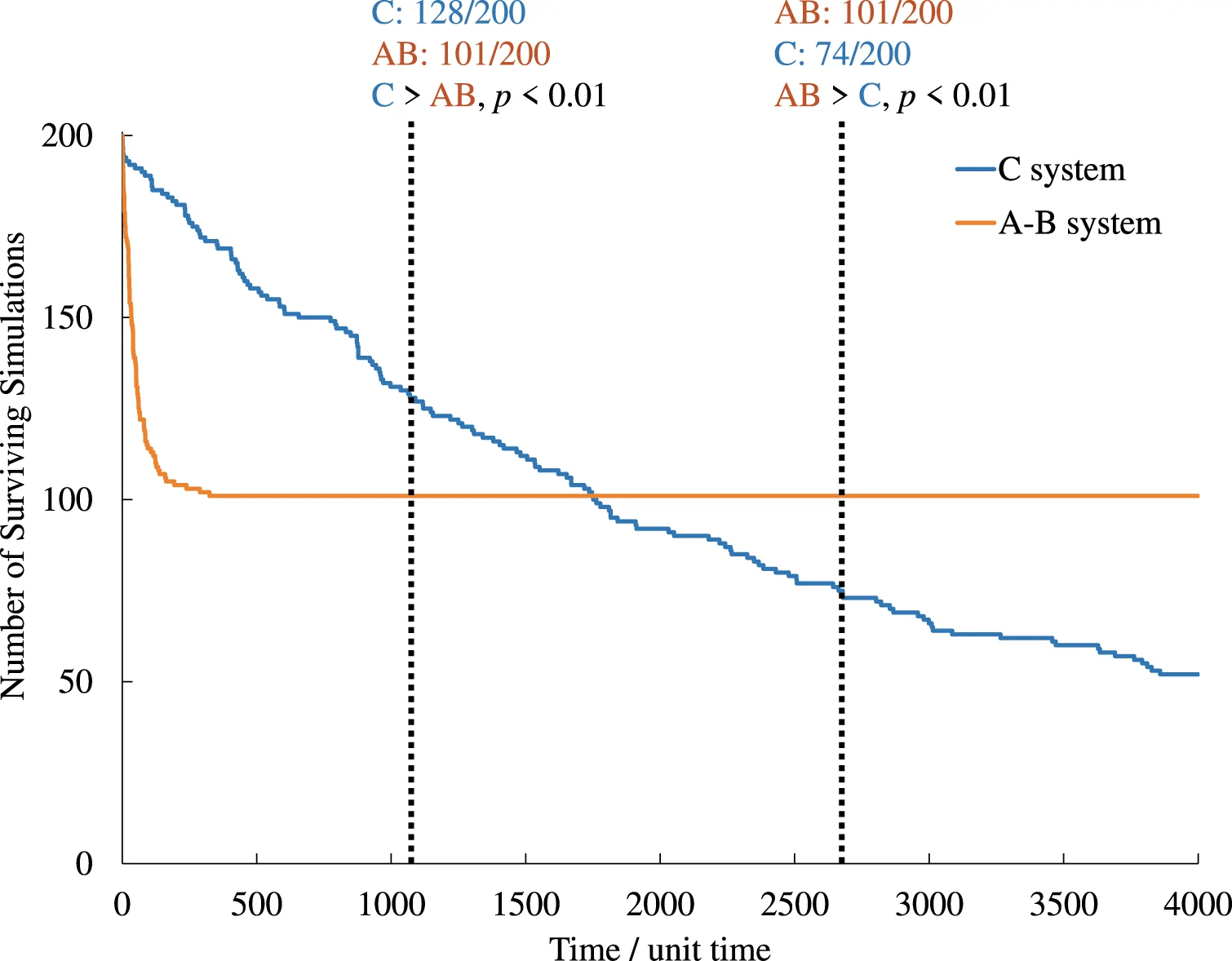
Compared with the side-reaction-free system (C system), the autocatalysis-side-reaction system (A–B system) may be less persistent in the short term but more persistent in the long term. For each of the two reaction systems, 200 replicate simulations were performed. The parameters were set as specified in supplementary material, table S1, case (d); all simulations start with 600 F molecules and 1 A or C molecule.

So far, our estimation of the persistence has been done by examining the dynamics of one autocatalytic system in one CSTR at a time. On the other hand, another common way to assess the persistence of self-sustaining systems is to let different systems compete for common resources in the same environment, such as bacterial competition assays. Therefore, one may wonder whether the results of direct competition between an A–B system and a C system in the same CSTR [32] are indeed consistent with the conclusions drawn from examining the two systems separately. Consequently, we consider the following reaction system composed of an A–B system and a C system competing for food F:

(2.10) $$\left\{ \begin{array}{l} A+F⇌2A\\ A⇌B\\ C+F⇌2C. \end{array}\right.$$

Simulating this reaction system in CSTRs, we found that the results of the competition do echo the patterns seen in figures 3e and 4. Figure 5 shows the survivorship curves of the A–B and C systems. Figure 5a,b uses the same parameter settings as figures 3e and 4, respectively, except that the simulations shown by figure 5a,b were initiated by adding 1 A molecule and 1 C molecule together into the same CSTR simultaneously. It is obvious that the shapes of the survivorship curves in figure 5a,b are highly similar to those of figures 3e and 4. These results confirm the validity of our method to estimate the persistence of autocatalytic systems.

**Figure 5.**
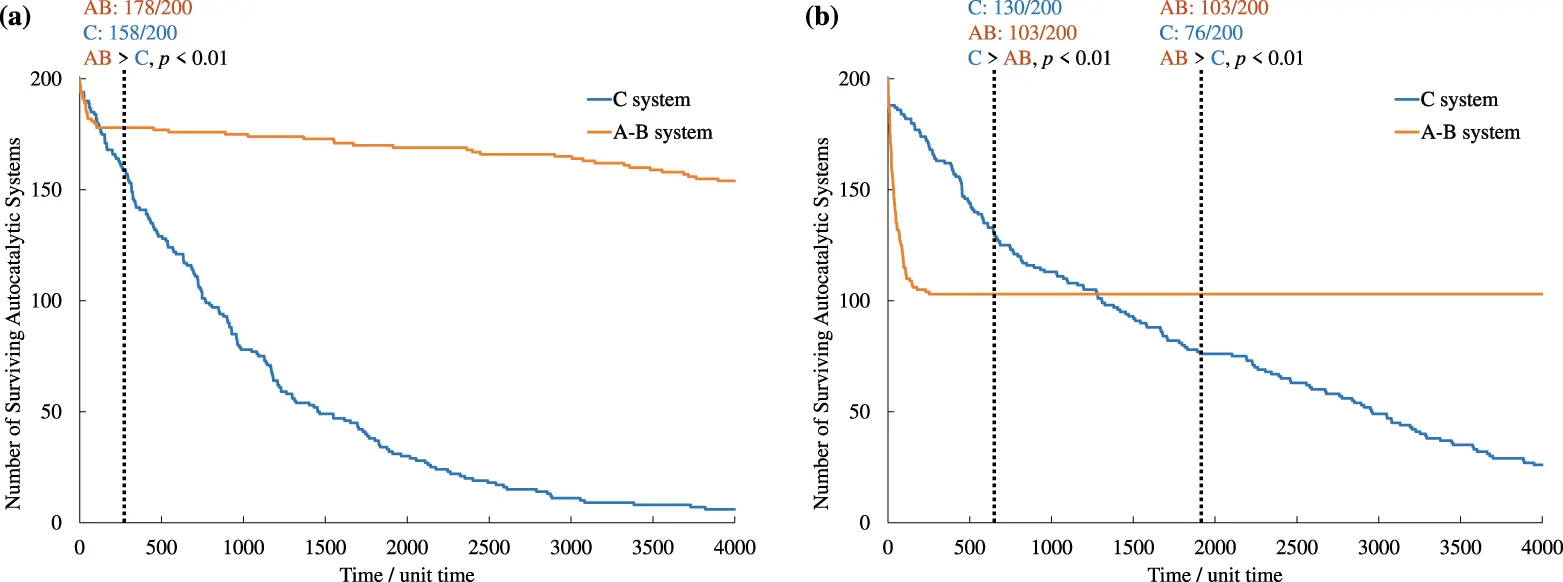
The results of competition between the autocatalysis-side-reaction system (A–B system) and the side-reaction-free system (C system) in the same CSTR. The A–B system and the C system are activated in the same CSTR to compete for the common food F. For each panel, 200 replicate simulations were performed. Fisher's exact test was used to calculate the *p*-values. (a) The parameter settings are the same as those of figure 3e; all simulations start with 500 F molecules, 1 A molecule, and 1 C molecule. (b) The parameter settings are the same as those of figure 4; all simulations start with 600 F molecules, 1 A molecule and 1 C molecule.

To summarize, for a reaction system consisting of the simplest autocatalytic reaction and the simplest side reaction, if one or both of the reactions are strictly irreversible, then the side reaction harms the persistence of the autocatalytic system. However, in the more realistic cases where the principle of microscopic reversibility holds, a side reaction can be harmful, neutral, beneficial, or harmful in the short term but beneficial in the long term, all depending on the kinetic parameters assumed.

### 2.4. A more complex reaction system: a two-reaction autocatalytic cycle with three side reactions

Thus far, we have only considered a simple reaction system consisting of one autocatalytic reaction and one side reaction. We have found that reversible side reactions slow down autocatalysis, which tends to decrease the concentration of direct autocatalysts, but also serve as a reservoir of indirect autocatalysts, which can facilitate system persistence. Given the principle of microscopic reversibility, the effects of side reactions ought to also apply to more complex reaction systems. As proof of principle, we conducted algebraic analysis of a reaction system consisting of a two-reaction autocatalytic cycle accompanied by three side reactions: A_1_ and A_2_ as direct autocatalysts, B_1_, B_2_ and B_3_ as side products (figure 6).

**Figure 6.**
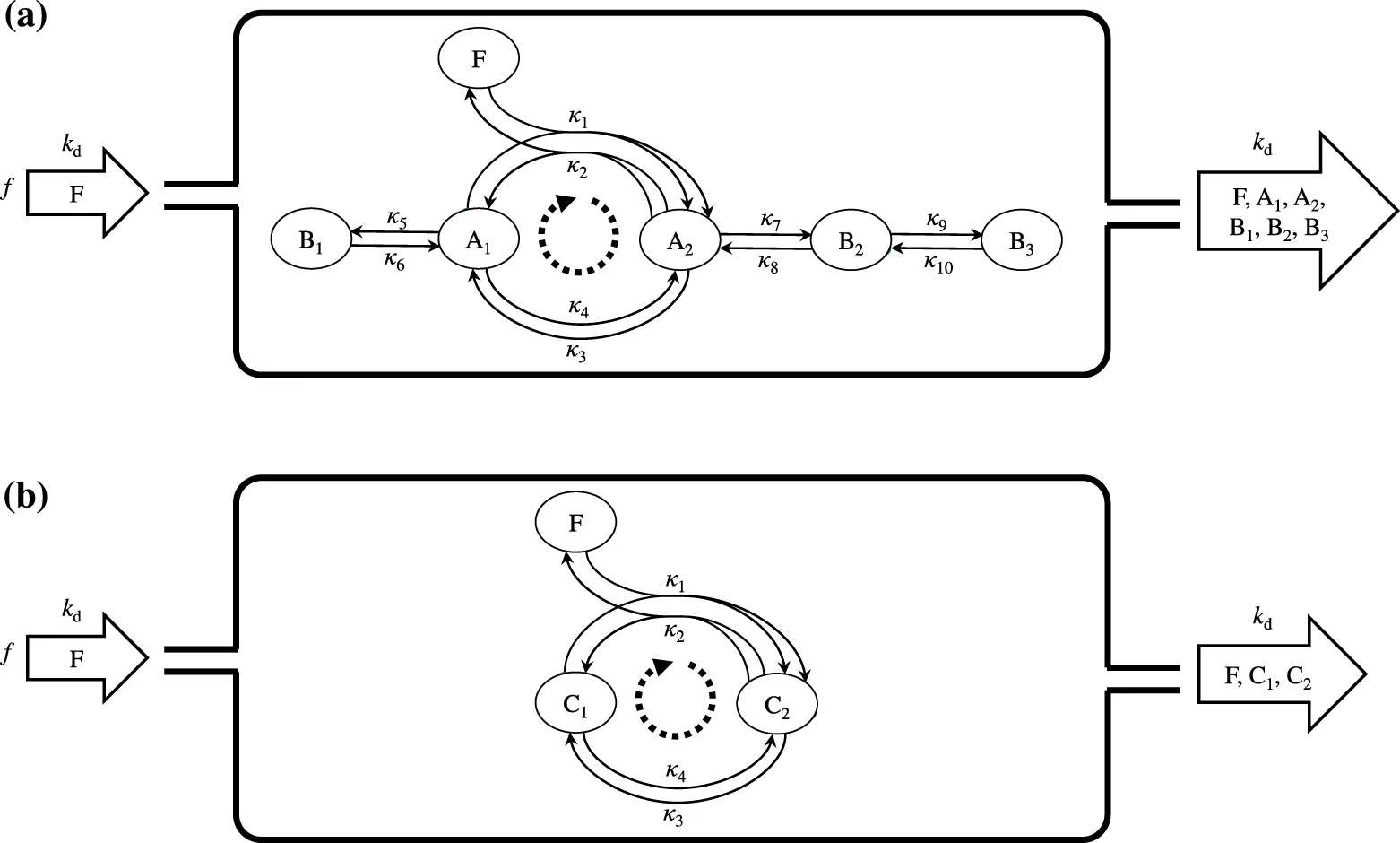
More complex autocatalytic systems. All reactions are reversible. The thick dotted arrow indicates the direction of autocatalysis. *f* is the concentration of F in the external food source; *k*_d_ is the dilution rate of the reactor; *κ*s are reaction rate constants. (a) A two-reaction autocatalytic cycle accompanied by three side reactions. A_1_ and A_2_ are direct autocatalysts; B_1_, B_2_ and B_3_ are side products. (b) A two-reaction autocatalytic cycle with no side reaction. C_1_ and C_2_ are direct autocatalysts.

As described more fully in supplementary material, appendix S6, by introducing a new parameter *Υ* that is similar to the *U* in §2.2, we can show that the influence of side reactions on the persistence of the autocatalytic system is as diverse as seen for the simple case studied above. Thus, we can find examples, such as those shown in figure 7 , where the total concentration of members of the autocatalytic system is higher or lower in the presence of the side reactions.

**Figure 7.**
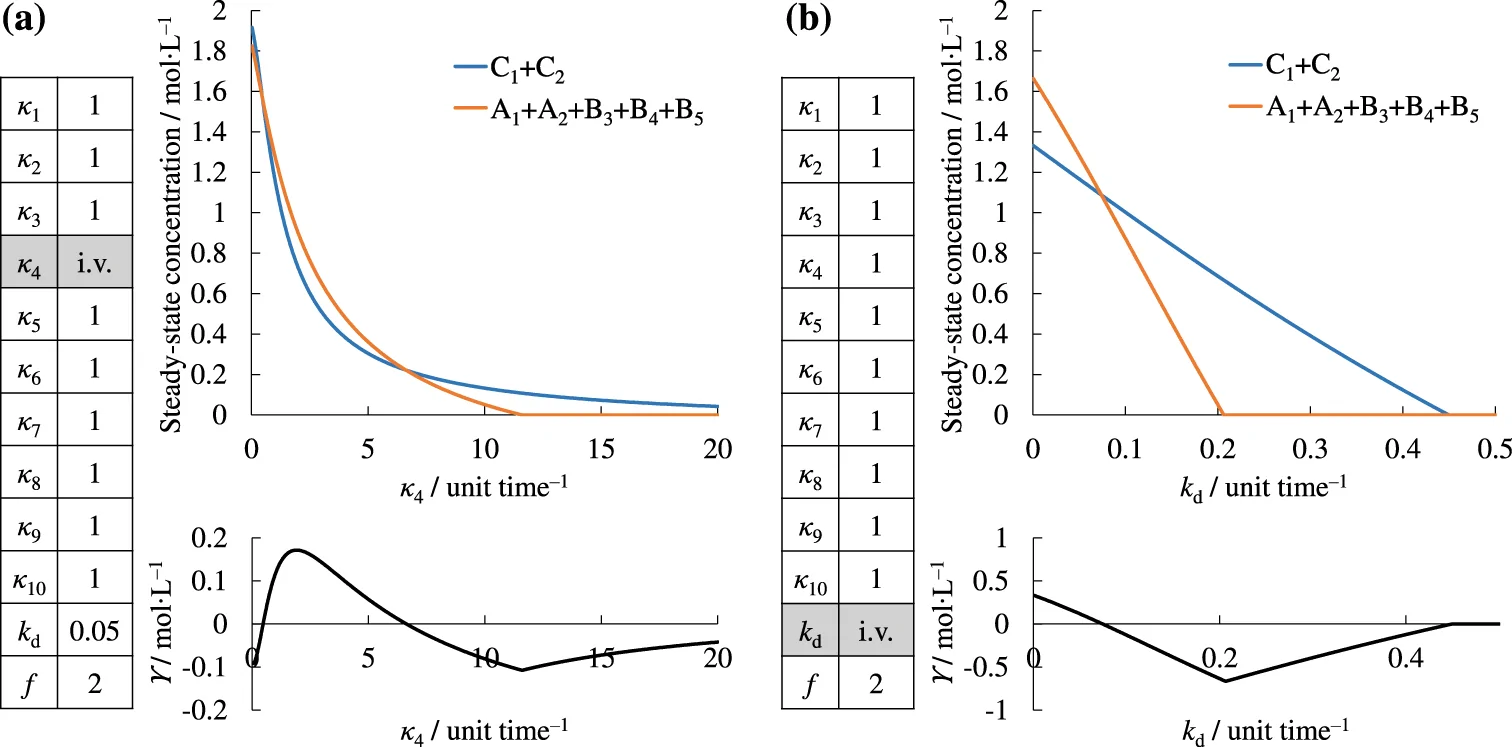
The influence of side reactions on the persistence of a complex autocatalytic system varies with parameter values. Reaction settings are as described in figure 6. Two examples of the association between *Υ*(*κ*_1_, *κ*_2_, *κ*_3_, *κ*_4_, *κ*_5_, *κ*_6_, *κ*_7_, *κ*_8_, *κ*_9_, *κ*_10_, *k*_d_, *f*) and parameter values are shown. *κ*s: reaction rate constants; unit: mol^–1^ l unit time^–1^ for *κ*_1_ and *κ*_2_, unit time^–1^ for *κ*_3_–*κ*_10_. *k*_d_, dilution rate; unit, unit time^–1^. *f*, food concentration of the external food source; unit: mol l^–1^. i.v., independent variable. (a) *κ*_4_ serves as the independent variable. (b) *k*_d_ serves as the independent variable.

## 3. Discussion

Our study responds to one of the most significant criticisms of metabolism-first theories that prebiotic autocatalytic metabolic cycles are inviable owing to side reactions, unless specialized catalysts are present that increase the autocatalytic rate constants relative to those of side reactions. We showed that even for a two-reaction system consisting of one second-order autocatalytic reaction and one first-order side reaction, the influence of the side reaction on the autocatalytic system is complex and highly parameter-dependent. Although strictly irreversible side reactions, which are mathematically conceivable but inconsistent with physical principles, are harmful, the effects of reversible side reactions are diverse, ranging from lethal to beneficial. Moreover, we also found cases in which a side reaction is harmful during the establishment phase of an autocatalytic system, but beneficial in the longer term. Further analysis showed that for a two-reaction autocatalytic cycle accompanied by three side reactions, the effects of side reactions are still diverse.

Although these findings were based on idealized and simple reaction networks, they make realistic chemical assumptions and would be expected to hold in practice. Notably, it was not necessary to assign additional beneficial effects to the products of side reactions for those side reactions to have a positive effect on the persistence of autocatalytic cycles. Therefore, the existence of side reactions consuming the members of autocatalytic cycles is not, in itself, a useful argument against metabolism-first theories.

Previous arguments for the lethal effects of side reactions on autocatalytic cycles often assume that reactions occur in closed or batch systems, which are most commonly studied by experimentalists. However, autocatalytic systems relevant to the emergence of life would have been open systems, experiencing an ongoing flux of food. So long as the food concentration and dilution rate are adequate, autocatalysis can be sustained in an open system, even when side reactions are present.

A second misunderstanding in some prior work is that treating side reactions as strictly irreversible is a valid approximation. In fact, allowing side reactions to be reversible, even if with highly asymmetric rate constants, means that side products become dual-role molecules: they have a negative effect by siphoning materials away from the core cycle but provide a reservoir of indirect autocatalysts that can sustain an activated autocatalytic system through environmental disturbances. The net effect of such side products depends on the relative weights of the two roles, which explains why we see such a wide range of effects of reversible side reactions.

There is a long-standing tradition in organic chemistry and chemical engineering of quantifying the effectiveness of a reaction or a series of sequential reactions based on yield, the percentage of reactants converted into the desired product. However, for prebiotic autocatalytic systems to persist, yield itself is not relevant. An autocatalytic system could plausibly persist with direct autocatalysts at concentrations below the limits of laboratory detection. What is at stake for metabolism-first theories is not creating an abundance of any one autocatalytic species, but the capacity of an autocatalytic motif to persist following its activation by the improbable introduction of a chemical seed (that is, one or a few molecules, introduced from elsewhere or generated by a very rare reaction [31]). Such seeding events cause the system to transition to a new dynamical steady state [30,32]—to go from a state in which the autocatalysts and side products are completely absent to one in which they all exist with non-zero concentration. This matters because it allows viable autocatalytic subnetworks to function as units of chemical memory, or chemes [33]. Like genes in biology, chemes can provide the basis for heritability and evolution [34].

Side reactions can also be beneficial beyond providing a reservoir of indirect seeds. For example, it is possible that some products of reversible side reactions (i.e. indirect autocatalysts) are more stable in the face of environmental disturbances than the direct autocatalysts. Because the presence of reversible side reactions expands the chemical space, it could be argued that the chances of finding at least one species that is tolerant of a particular kind of environmental disturbance are greater when there are more side-reaction pathways. Thus, side reactions may serve to make an autocatalytic system more robust to environmental stress.

Our results are consistent with the idea that, owing to side reactions and other factors, autocatalytic cycles could differ in their capacity to persist once activated, which is sufficient for a primordial mode of adaptive evolution [31,34]. This would occur when there are multiple, spatially localized systems that differ in which chemes are active and when selection favours those that are better able to persist and spread to other locations. Although this idea remains controversial, our demonstration that autocatalytic cycles, and thus chemes, are potentially robust even if they trigger side reactions, expands the set of chemical contexts in which metabolism-first models can viably explain evolutionary complexification before the emergence of specialized catalysts or templated replication.

## 4. Material and methods

Simulations of stochastic chemical kinetics were performed using the Gillespie algorithm [26–28]. The Python scripts used for simulations can be found in the supplementary material.

## Supplementary Material



## Data Availability

The code (Python scripts) and Excel files can be found in the supplementary material; these files can be used to reproduce the results shown in (figures 2–5) and figure 7. Supplementary material is available online [35].
