# Supplementary material for: The diverse effects of side reactions on the persistence of autocatalytic cycles
Source: J R Soc Interface. 2026 Oct 14;23(243):20260308. doi: 10.1098/rsif.2026.0308 (PMC13682562; doi:10.1098/rsif.2026.0308)
Supplement: Supplementary file 1 [file rsif.2026.0308.zip › rsif.2026.0308/rsif20260308_si_001.docx]

**Supplemental Information for:**

**The diverse effects of side reactions on the persistence of autocatalytic cycles**

**Authors & Affiliations**

Zhen Peng^1,2^*, Emily Spencer^2^, David A. Baum^2,3^*

^1^State Key Laboratory of Bioreactor Engineering, School of Biotechnology, East China University of Science and Technology, Shanghai, 200237, China

^2^Wisconsin Institute for Discovery, University of Wisconsin–Madison, Madison, WI 53706, USA

^3^Department of Botany, University of Wisconsin–Madison, Madison, WI 53706, USA

*Correspondence to: [zhen.peng@ecust.edu.cn](mailto:zhen.peng@ecust.edu.cn), [peng.zhen2002@outlook.com](mailto:peng.zhen2002@outlook.com), [dbaum@wisc.edu](mailto:dbaum@wisc.edu)

**Appendix 1: Autocatalytic reaction without side reaction**

This appendix relates to Section 2.2.

The changes in the concentrations of C and F over time are described by

$$\begin{aligned} \left\{ \begin{matrix} \frac{d\left[ C \right]}{dt}=-k_{d}\left[ C \right]+k_{1}\left[ C \right]\left[ F \right]-k_{2}\left[ C \right]^{2} \\ \frac{d\left[ F \right]}{dt}=k_{d}f-k_{d}\left[ F \right]-k_{1}\left[ C \right]\left[ F \right]+k_{2}\left[ C \right]^{2} \end{matrix} \right.\#\left( S1 \right) \end{aligned}$$

Let

$$\begin{aligned} \left\{ \begin{matrix} 0=-k_{d}\left[ C \right]_{s}+k_{1}\left[ C \right]_{s}\left[ F \right]_{s}-k_{2}\left[ C \right]_{s}^{2} \\ 0=k_{d}f-k_{d}\left[ F \right]_{s}-k_{1}\left[ C \right]_{s}\left[ F \right]_{s}+k_{2}\left[ C \right]_{s}^{2} \end{matrix} \right.\#\left( S2 \right) \end{aligned}$$

Then the steady-state concentrations are either

$$\begin{aligned} \left\{ \begin{matrix} \left[ C \right]_{s}=0 \\ \left[ F \right]_{s}=f \end{matrix} \right.\#\left( 2.5 \right) \end{aligned}$$

or

$$\begin{aligned} \left\{ \begin{matrix} \left[ C \right]_{s}=\frac{k_{1}f-k_{d}}{k_{1}+k_{2}} \\ \left[ F \right]_{s}=f-\left[ C \right]_{s} \end{matrix} \right.\#\left( 2.6 \right) \end{aligned}$$

**Appendix 2: Reversible autocatalytic reaction and side reaction**

This appendix relates to Section 2.2.

The changes in the concentrations of A, B, and F over time are described by

$$\begin{aligned} \left\{ \begin{matrix} \frac{d\left[ A \right]}{dt}=-k_{d}\left[ A \right]+k_{1}\left[ A \right]\left[ F \right]-k_{2}\left[ A \right]^{2}-k_{3}\left[ A \right]+k_{4}\left[ B \right] \\ \frac{d\left[ B \right]}{dt}=-k_{d}\left[ B \right]+k_{3}\left[ A \right]-k_{4}\left[ B \right] \\ \frac{d\left[ F \right]}{dt}=k_{d}f-k_{d}\left[ F \right]-k_{1}\left[ A \right]\left[ F \right]+k_{2}\left[ A \right]^{2} \end{matrix} \right.\#\left( S3 \right) \end{aligned}$$

Let

$$\begin{aligned} \left\{ \begin{matrix} 0=-k_{d}\left[ A \right]_{s}+k_{1}\left[ A \right]_{s}\left[ F \right]_{s}-k_{2}\left[ A \right]_{s}^{2}-k_{3}\left[ A \right]_{s}+k_{4}\left[ B \right]_{s} \\ 0=-k_{d}\left[ B \right]_{s}+k_{3}\left[ A \right]_{s}-k_{4}\left[ B \right]_{s} \\ 0=k_{d}f-k_{d}\left[ F \right]_{s}-k_{1}\left[ A \right]_{s}\left[ F \right]_{s}+k_{2}\left[ A \right]_{s}^{2} \end{matrix} \right.\#\left( S4 \right) \end{aligned}$$

Then the steady-state concentrations are either

$$\begin{aligned} \left\{ \begin{matrix} \left[ A \right]_{s}=0 \\ \left[ B \right]_{s}=0 \\ \left[ F \right]_{s}=f \end{matrix} \right.\#\left( 2.1 \right) \end{aligned}$$

or

$$\begin{aligned} \left\{ \begin{matrix} \left[ A \right]_{s}=\frac{k_{1}k_{4}f+k_{d}k_{1}f-k_{d}k_{3}-k_{d}k_{4}-k_{d}^{2}}{k_{1}k_{3}+k_{1}k_{4}+k_{2}k_{4}+k_{d}k_{1}+k_{d}k_{2}} \\ \left[ B \right]_{s}=\frac{k_{3}}{k_{d}+k_{4}}\left[ A \right]_{s} \\ \left[ F \right]_{s}=f-\frac{k_{d}+k_{3}+k_{4}}{k_{d}+k_{4}}\left[ A \right]_{s} \end{matrix} \right.\#\left( 2.2 \right) \end{aligned}$$

According to Equations (2.2) and (2.6), we have

$$\begin{aligned} \begin{matrix} U\left( k_{1},k_{2},k_{3},k_{4},k_{d},f \right)= \\ \max\left( 0, \frac{k_{d}+k_{3}+k_{4}}{k_{d}+k_{4}}\cdot\frac{k_{1}k_{4}f+k_{d}k_{1}f-k_{d}k_{3}-k_{d}k_{4}-k_{d}^{2}}{k_{1}k_{3}+k_{1}k_{4}+k_{2}k_{4}+k_{d}k_{1}+k_{d}k_{2}} \right) \\ -max\left( 0, \frac{k_{1}f-k_{d}}{k_{1}+k_{2}} \right) \end{matrix}\#\left( 2.9 \right) \end{aligned}$$

Equation (2.9) was used to generate the data for Fig. 2.

**Appendix 3: Strictly irreversible side reaction**

This appendix corresponds to Section 2.2.

Since the side reaction is irreversible, *k*_4_ = 0. The changes in the concentrations of A, B, and F over time are described by

$$\begin{aligned} \left\{ \begin{matrix} \frac{d\left[ A \right]}{dt}=-k_{d}\left[ A \right]+k_{1}\left[ A \right]\left[ F \right]-k_{2}\left[ A \right]^{2}-k_{3}\left[ A \right] \\ \frac{d\left[ B \right]}{dt}=-k_{d}\left[ B \right]+k_{3}\left[ A \right] \\ \frac{d\left[ F \right]}{dt}=k_{d}f-k_{d}\left[ F \right]-k_{1}\left[ A \right]\left[ F \right]+k_{2}\left[ A \right]^{2} \end{matrix} \right.\#\left( S5 \right) \end{aligned}$$

Denoting the steady-state concentrations of A, B, and F by [A]_s_, [B]_s_, and [F]_s_, respectively, we get

$$\begin{aligned} \left\{ \begin{matrix} 0=-k_{d}\left[ A \right]_{s}+k_{1}\left[ A \right]_{s}\left[ F \right]_{s}-k_{2}\left[ A \right]_{s}^{2}-k_{3}\left[ A \right]_{s} \\ 0=-k_{d}\left[ B \right]_{s}+k_{3}\left[ A \right]_{s} \\ 0=k_{d}f-k_{d}\left[ F \right]_{s}-k_{1}\left[ A \right]_{s}\left[ F \right]_{s}+k_{2}\left[ A \right]_{s}^{2} \end{matrix} \right.\#\left( S6 \right) \end{aligned}$$

which results in two possible sets of steady-state concentrations:

$$\begin{aligned} \left\{ \begin{matrix} \left[ A \right]_{s}=0 \\ \left[ B \right]_{s}=0 \\ \left[ F \right]_{s}=f \end{matrix} \right.\#\left( 2.1 \right) \end{aligned}$$

or

$$\begin{aligned} \left\{ \begin{matrix} \left[ A \right]_{s}=\frac{k_{d}k_{1}f-k_{d}k_{3}-k_{d}^{2}}{k_{1}k_{3}+k_{d}k_{1}+k_{d}k_{2}} \\ \left[ B \right]_{s}=\frac{k_{3}}{k_{d}}\left[ A \right]_{s} \\ \left[ F \right]_{s}=f-\frac{k_{d}+k_{3}}{k_{d}}\left[ A \right]_{s} \end{matrix} \right.\#\left( 2.3 \right) \end{aligned}$$

Then let [A]_s_ > 0, we get

$$\begin{aligned} \frac{k_{d}k_{1}f-k_{d}k_{3}-k_{d}^{2}}{k_{1}k_{3}+k_{d}k_{1}+k_{d}k_{2}}>0\#\left( S7 \right) \end{aligned}$$

Since the reactor is open (i.e., *k*_d_ > 0), we have

$$\begin{aligned} k_{1}f-k_{3}-k_{d}>0\#\left( S8 \right) \end{aligned}$$

which yields

$$\begin{aligned} k_{1}>\frac{k_{3}+k_{d}}{f}\#\left( 2.4 \right) \end{aligned}$$

For the case of non-extinction, according to Equations (2.3) and (2.6), we have

$$\begin{aligned} \left[ A \right]_{s}-\left[ C \right]_{s}=\frac{k_{d}k_{1}f-k_{d}k_{3}-k_{d}^{2}}{k_{1}k_{3}+k_{d}k_{1}+k_{d}k_{2}}-\frac{k_{1}f-k_{d}}{k_{1}+k_{2}} \#\left( S9 \right) \end{aligned}$$

$$\begin{aligned} \left[ A \right]_{s}-\left[ C \right]_{s}=\frac{-k_{d}k_{2}k_{3}-k_{1}^{2}k_{3}f}{\left( k_{1}k_{3}+k_{d}k_{1}+k_{d}k_{2} \right)\left( k_{1}+k_{2} \right)}<0\#\left( S10 \right) \end{aligned}$$

In addition, if [C]_s_ = 0, then *k*_1_*f* – *k*_d_ ≤ 0 ⇒ *k*_d_(*k*_1_*f* – *k*_d_) ≤ 0 ⇒ *k*_d_(*k*_1_*f* – *k*_d_) – *k*_d_*k*_3_ < 0, and thus [A]_s_ must be 0.

**Appendix 4: Strictly irreversible autocatalytic reaction with reversible side reaction**

This appendix relates to Section 2.2.

Since the autocatalytic reaction is irreversible, *k*_2_ = 0. The changes in the concentrations of A, B, and F over time are described by

$$\begin{aligned} \left\{ \begin{matrix} \frac{d\left[ A \right]}{dt}=-k_{d}\left[ A \right]+k_{1}\left[ A \right]\left[ F \right]-k_{3}\left[ A \right]+k_{4}\left[ B \right] \\ \frac{d\left[ B \right]}{dt}=-k_{d}\left[ B \right]+k_{3}\left[ A \right]-k_{4}\left[ B \right] \\ \frac{d\left[ F \right]}{dt}=k_{d}f-k_{d}\left[ F \right]-k_{1}\left[ A \right]\left[ F \right] \end{matrix} \right.\#\left( S11 \right) \end{aligned}$$

Let

$$\begin{aligned} \left\{ \begin{matrix} 0=-k_{d}\left[ A \right]_{s}+k_{1}\left[ A \right]_{s}\left[ F \right]_{s}-k_{3}\left[ A \right]_{s}+k_{4}\left[ B \right]_{s} \\ 0=-k_{d}\left[ B \right]_{s}+k_{3}\left[ A \right]_{s}-k_{4}\left[ B \right]_{s} \\ 0=k_{d}f-k_{d}\left[ F \right]_{s}-k_{1}\left[ A \right]_{s}\left[ F \right]_{s} \end{matrix} \right.\#\left( S12 \right) \end{aligned}$$

Then the steady-state concentrations are either

$$\begin{aligned} \left\{ \begin{matrix} \left[ A \right]_{s}=0 \\ \left[ B \right]_{s}=0 \\ \left[ F \right]_{s}=f \end{matrix} \right.\#\left( 2.1 \right) \end{aligned}$$

or

$$\begin{aligned} \left\{ \begin{matrix} \left[ A \right]_{s}=\frac{k_{1}k_{4}f+k_{d}k_{1}f-k_{d}k_{3}-k_{d}k_{4}-k_{d}^{2}}{k_{1}k_{3}+k_{1}k_{4}+k_{d}k_{1}} \\ \left[ B \right]_{s}=\frac{k_{3}}{k_{d}+k_{4}}\left[ A \right]_{s} \\ \left[ F \right]_{s}=f-\frac{k_{d}+k_{3}+k_{4}}{k_{d}+k_{4}}\left[ A \right]_{s} \end{matrix} \right.\#\left( 2.7 \right) \end{aligned}$$

For the case of non-extinction, according to Equations (2.6) and (2.7) (note that *k*_2_ = 0), we have

$$\begin{aligned} \left[ A \right]_{s}+\left[ B \right]_{s}-\left[ C \right]_{s}=\frac{k_{d}+k_{3}+k_{4}}{k_{d}+k_{4}}\cdot\frac{k_{1}k_{4}f+k_{d}k_{1}f-k_{d}k_{3}-k_{d}k_{4}-k_{d}^{2}}{k_{1}k_{3}+k_{1}k_{4}+k_{d}k_{1}}-\frac{k_{1}f-k_{d}}{k_{1}} \#\left( S13 \right) \end{aligned}$$

$$\begin{aligned} \left[ A \right]_{s}+\left[ B \right]_{s}-\left[ C \right]_{s}= \frac{-k_{d}k_{3}}{k_{d}k_{1}+k_{1}k_{4}}<0\#\left( 2.8 \right) \end{aligned}$$

Therefore, the side reaction harms the persistence of the autocatalytic system.

In addition, if [C]_s_ = 0, then *k*_1_*f* – *k*_d_ ≤ 0 ⇒ (*k*_4_ + *k*_d_)(*k*_1_*f* – *k*_d_) ≤ 0 ⇒ (*k*_4_ + *k*_d_)(*k*_1_*f* – *k*_d_) – *k*_d_*k*_3_ < 0, and thus both [A]_s_ and [B]_s_ must be 0.

**Appendix 5: Competition between the autocatalysis-side-reaction system (A-B system) and the side-reaction-free system (C system) in the same CSTR**

This appendix relates to Section 2.3.

Since the autocatalytic reaction

The competition between the A-B system and the C system in the same CSTR is described by

$$\begin{aligned} \left\{ \begin{matrix} \frac{d\left[ A \right]}{dt}=-k_{d}\left[ A \right]+k_{1}\left[ A \right]\left[ F \right]-k_{2}\left[ A \right]^{2}-k_{3}\left[ A \right]+k_{4}\left[ B \right] \\ \frac{d\left[ B \right]}{dt}=-k_{d}\left[ B \right]+k_{3}\left[ A \right]-k_{4}\left[ B \right] \\ \frac{d\left[ C \right]}{dt}=-k_{d}\left[ C \right]+k_{1}\left[ C \right]\left[ F \right]-k_{2}\left[ C \right]^{2} \\ \frac{d\left[ F \right]}{dt}=k_{d}f-k_{d}\left[ F \right]-k_{1}\left[ A \right]\left[ F \right]+k_{2}\left[ A \right]^{2}-k_{1}\left[ C \right]\left[ F \right]+k_{2}\left[ C \right]^{2} \end{matrix} \right.\#\left( S14 \right) \end{aligned}$$

Let

$$\begin{aligned} \left\{ \begin{matrix} 0=-k_{d}\left[ A \right]_{s}+k_{1}\left[ A \right]_{s}\left[ F \right]_{s}-k_{2}\left[ A \right]_{s}^{2}-k_{3}\left[ A \right]_{s}+k_{4}\left[ B \right]_{s} \\ 0=-k_{d}\left[ B \right]_{s}+k_{3}\left[ A \right]_{s}-k_{4}\left[ B \right]_{s} \\ 0=-k_{d}\left[ C \right]_{s}+k_{1}\left[ C \right]_{s}\left[ F \right]_{s}-k_{2}\left[ C \right]_{s}^{2} \\ 0=k_{d}f-k_{d}\left[ F \right]_{s}-k_{1}\left[ A \right]_{s}\left[ F \right]_{s}+k_{2}\left[ A \right]_{s}^{2}-k_{1}\left[ C \right]_{s}\left[ F \right]_{s}+k_{2}\left[ C \right]_{s}^{2} \end{matrix} \right.\#\left( S15 \right) \end{aligned}$$

Then the steady-state concentrations of all chemical species can be computed manually or using software (e.g., we used Mathematica™ 13.3). Since the solutions are complicated, we decided to provide them in a format that can be easily imported to software like Microsoft Excel and Python, which should help readers replicate our results. Note that if Python is going to be used, the “^” in the solution needs to be replaced by “**”.

Solution 1 (S16) (deactivated A-B system and C system):

[A]_s_ = 0

[B]_s_ = 0

[C]_s_ = 0

[F]_s_ = f

Solution 2 (S17) (deactivated A-B system; activated C system):

[A]_s_ = 0

[B]_s_ = 0

[C]_s_ = (f*k1 - kd)/(k1 + k2)

[F]_s_ = (f*k2 + kd)/(k1 + k2)

Solution 3 (S18) (activated A-B system survives; deactivated C system):

[A]_s_ = (f*k1*(k4 + kd) - kd*(k3 + k4 + kd))/(k2*(k4 + kd) + k1*(k3 + k4 + kd))

[B]_s_ = (f*k1*k3*(k4 + kd) - k3*kd*(k3 + k4 + kd))/((k4 + kd)*(k2*(k4 + kd) + k1*(k3 + k4 + kd)))

[C]_s_ = 0

[F]_s_ = (f*k2*(k4 + kd)^2 + kd*(k3 + k4 + kd)^2)/((k4 + kd)*(k2*(k4 + kd) + k1*(k3 + k4 + kd)))

Solution 4 (S19) (activated A-B system and C system):

[A]_s_ = (f*k1*k2*(k4 + kd) - kd*(k1*k3 + k2*(k3 + k4 + kd)))/(k2*(k2*(k4 + kd) + k1*(k3 + 2*(k4 + kd))))

[B]_s_ = -((k3*(k1*k3*kd - f*k1*k2*(k4 + kd) + k2*kd*(k3 + k4 + kd)))/(k2*(k4 + kd)*(k2*(k4 + kd) + k1*(k3 + 2*(k4 + kd)))))

[C]_s_ = (f*k1*k2*(k4 + kd)^2 - k2*kd*(k4 + kd)^2 + k1*k3*kd*(k3 + k4 + kd))/(k2*(k4 + kd)*(k2*(k4 + kd) + k1*(k3 + 2*(k4 + kd))))

[F]_s_ = (f*k2*(k4 + kd)^2 + kd*(k3^2 + 2*k3*(k4 + kd) + 2*(k4 + kd)^2))/((k4 + kd)*(k2*(k4 + kd) + k1*(k3 + 2*(k4 + kd))))

**Appendix 6: A more complex reaction system**

This appendix corresponds to Section 2.4.

We define

$$\begin{aligned} \Upsilon\left( \kappa_{1},\kappa_{2},\kappa_{3},\kappa_{4},\kappa_{5},\kappa_{6},\kappa_{7},\kappa_{8},\kappa_{9},\kappa_{10},k_{d},f \right)=\sum_{i=1}^{2} \left[ A_{i} \right]_{s}+\sum_{j=1}^{3} \left[ B_{j} \right]_{s}-\sum_{i=1}^{2} \left[ C_{i} \right]_{s}\#\left( S20 \right) \end{aligned}$$

Since there are 10 reaction rate constants and 6 chemical species, the differential equation system is complicated. To improve readability, we show the differential equations that describe the changes in the concentrations of A*_i_*’s, B*_j_*’s, and F over time as a table:

 (S21)

By letting all equations equal zero, the steady-state concentrations of all chemical species can be computed manually or using software (e.g., we used Mathematica™ 13.3). Like Appendix 5, we decided to provide the solutions in a format that can be easily imported to software like Microsoft Excel and Python, which should help readers replicate our results. Note that if Python is going to be used, the “^” in the solution needs to be replaced by “**”.

Solution 1 (S22) (extinction):

[A_1_]_s_ = 0

[A_2_]_s_ = 0

[B_1_]_s_ = 0

[B_2_]_s_ = 0

[B_3_]_s_ = 0

[F]_s_ = f

Solution 2 (S23) (survival):

[A_1_]_s_ = ((Kappa6+kd)*(Kappa10*((-Kappa3)*(Kappa8+kd)+kd*(Kappa7+Kappa8+kd))+kd*(Kappa7*(Kappa9+kd)-Kappa3*(Kappa8+Kappa9+kd)+kd*(Kappa8+Kappa9+kd)))*(f*Kappa1*(Kappa6+kd)*(Kappa10*((-Kappa3)*(Kappa8+kd)+kd*(Kappa7+Kappa8+kd))+kd*(Kappa7*(Kappa9+kd)-Kappa3*(Kappa8+Kappa9+kd)+kd*(Kappa8+Kappa9+kd)))+kd*(Kappa4*(Kappa6+kd)*(Kappa7*(Kappa9+kd)+kd*(Kappa8+Kappa9+kd))+kd*(Kappa5+Kappa6+kd)*(Kappa7*(Kappa9+kd)+Kappa3*(Kappa8+Kappa9+kd)+kd*(Kappa8+Kappa9+kd))+Kappa10*(Kappa4*(Kappa6+kd)*(Kappa7+Kappa8+kd)+(Kappa5+Kappa6+kd)*(Kappa3*(Kappa8+kd)+kd*(Kappa7+Kappa8+kd))))))/(Kappa2*(Kappa4*(Kappa6+kd)+2*kd*(Kappa5+Kappa6+kd))^2*(Kappa10*(Kappa8+kd)+kd*(Kappa8+Kappa9+kd))^2-Kappa1*(Kappa6+kd)*(Kappa10*((-Kappa3)*(Kappa8+kd)+kd*(Kappa7+Kappa8+kd))+kd*(Kappa7*(Kappa9+kd)-Kappa3*(Kappa8+Kappa9+kd)+kd*(Kappa8+Kappa9+kd)))*(Kappa4*(Kappa6+kd)*(Kappa7*(Kappa9+kd)+kd*(Kappa8+Kappa9+kd))+kd*(Kappa5+Kappa6+kd)*(Kappa7*(Kappa9+kd)+Kappa3*(Kappa8+Kappa9+kd)+kd*(Kappa8+Kappa9+kd))+Kappa10*(Kappa4*(Kappa6+kd)*(Kappa7+Kappa8+kd)+(Kappa5+Kappa6+kd)*(Kappa3*(Kappa8+kd)+kd*(Kappa7+Kappa8+kd)))))

[A_2_]_s_ = -(((Kappa4*(Kappa6+kd)+2*kd*(Kappa5+Kappa6+kd))*(Kappa10*(Kappa8+kd)+kd*(Kappa8+Kappa9+kd))*(f*Kappa1*(Kappa6+kd)*(Kappa10*((-Kappa3)*(Kappa8+kd)+kd*(Kappa7+Kappa8+kd))+kd*(Kappa7*(Kappa9+kd)-Kappa3*(Kappa8+Kappa9+kd)+kd*(Kappa8+Kappa9+kd)))+kd*(Kappa4*(Kappa6+kd)*(Kappa7*(Kappa9+kd)+kd*(Kappa8+Kappa9+kd))+kd*(Kappa5+Kappa6+kd)*(Kappa7*(Kappa9+kd)+Kappa3*(Kappa8+Kappa9+kd)+kd*(Kappa8+Kappa9+kd))+Kappa10*(Kappa4*(Kappa6+kd)*(Kappa7+Kappa8+kd)+(Kappa5+Kappa6+kd)*(Kappa3*(Kappa8+kd)+kd*(Kappa7+Kappa8+kd))))))/(Kappa2*(Kappa4*(Kappa6+kd)+2*kd*(Kappa5+Kappa6+kd))^2*(Kappa10*(Kappa8+kd)+kd*(Kappa8+Kappa9+kd))^2-Kappa1*(Kappa6+kd)*(Kappa10*((-Kappa3)*(Kappa8+kd)+kd*(Kappa7+Kappa8+kd))+kd*(Kappa7*(Kappa9+kd)-Kappa3*(Kappa8+Kappa9+kd)+kd*(Kappa8+Kappa9+kd)))*(Kappa4*(Kappa6+kd)*(Kappa7*(Kappa9+kd)+kd*(Kappa8+Kappa9+kd))+kd*(Kappa5+Kappa6+kd)*(Kappa7*(Kappa9+kd)+Kappa3*(Kappa8+Kappa9+kd)+kd*(Kappa8+Kappa9+kd))+Kappa10*(Kappa4*(Kappa6+kd)*(Kappa7+Kappa8+kd)+(Kappa5+Kappa6+kd)*(Kappa3*(Kappa8+kd)+kd*(Kappa7+Kappa8+kd))))))

[B_1_]_s_ = -((Kappa5*(Kappa10*(Kappa3*(Kappa8+kd)-kd*(Kappa7+Kappa8+kd))-kd*(Kappa7*(Kappa9+kd)-Kappa3*(Kappa8+Kappa9+kd)+kd*(Kappa8+Kappa9+kd)))*(f*Kappa1*(Kappa6+kd)*(Kappa10*((-Kappa3)*(Kappa8+kd)+kd*(Kappa7+Kappa8+kd))+kd*(Kappa7*(Kappa9+kd)-Kappa3*(Kappa8+Kappa9+kd)+kd*(Kappa8+Kappa9+kd)))+kd*(Kappa4*(Kappa6+kd)*(Kappa7*(Kappa9+kd)+kd*(Kappa8+Kappa9+kd))+kd*(Kappa5+Kappa6+kd)*(Kappa7*(Kappa9+kd)+Kappa3*(Kappa8+Kappa9+kd)+kd*(Kappa8+Kappa9+kd))+Kappa10*(Kappa4*(Kappa6+kd)*(Kappa7+Kappa8+kd)+(Kappa5+Kappa6+kd)*(Kappa3*(Kappa8+kd)+kd*(Kappa7+Kappa8+kd))))))/(Kappa2*(Kappa4*(Kappa6+kd)+2*kd*(Kappa5+Kappa6+kd))^2*(Kappa10*(Kappa8+kd)+kd*(Kappa8+Kappa9+kd))^2-Kappa1*(Kappa6+kd)*(Kappa10*((-Kappa3)*(Kappa8+kd)+kd*(Kappa7+Kappa8+kd))+kd*(Kappa7*(Kappa9+kd)-Kappa3*(Kappa8+Kappa9+kd)+kd*(Kappa8+Kappa9+kd)))*(Kappa4*(Kappa6+kd)*(Kappa7*(Kappa9+kd)+kd*(Kappa8+Kappa9+kd))+kd*(Kappa5+Kappa6+kd)*(Kappa7*(Kappa9+kd)+Kappa3*(Kappa8+Kappa9+kd)+kd*(Kappa8+Kappa9+kd))+Kappa10*(Kappa4*(Kappa6+kd)*(Kappa7+Kappa8+kd)+(Kappa5+Kappa6+kd)*(Kappa3*(Kappa8+kd)+kd*(Kappa7+Kappa8+kd))))))

[B_2_]_s_ = -((Kappa7*(Kappa10+kd)*(Kappa4*(Kappa6+kd)+2*kd*(Kappa5+Kappa6+kd))*(f*Kappa1*(Kappa6+kd)*(Kappa10*((-Kappa3)*(Kappa8+kd)+kd*(Kappa7+Kappa8+kd))+kd*(Kappa7*(Kappa9+kd)-Kappa3*(Kappa8+Kappa9+kd)+kd*(Kappa8+Kappa9+kd)))+kd*(Kappa4*(Kappa6+kd)*(Kappa7*(Kappa9+kd)+kd*(Kappa8+Kappa9+kd))+kd*(Kappa5+Kappa6+kd)*(Kappa7*(Kappa9+kd)+Kappa3*(Kappa8+Kappa9+kd)+kd*(Kappa8+Kappa9+kd))+Kappa10*(Kappa4*(Kappa6+kd)*(Kappa7+Kappa8+kd)+(Kappa5+Kappa6+kd)*(Kappa3*(Kappa8+kd)+kd*(Kappa7+Kappa8+kd))))))/(Kappa2*(Kappa4*(Kappa6+kd)+2*kd*(Kappa5+Kappa6+kd))^2*(Kappa10*(Kappa8+kd)+kd*(Kappa8+Kappa9+kd))^2-Kappa1*(Kappa6+kd)*(Kappa10*((-Kappa3)*(Kappa8+kd)+kd*(Kappa7+Kappa8+kd))+kd*(Kappa7*(Kappa9+kd)-Kappa3*(Kappa8+Kappa9+kd)+kd*(Kappa8+Kappa9+kd)))*(Kappa4*(Kappa6+kd)*(Kappa7*(Kappa9+kd)+kd*(Kappa8+Kappa9+kd))+kd*(Kappa5+Kappa6+kd)*(Kappa7*(Kappa9+kd)+Kappa3*(Kappa8+Kappa9+kd)+kd*(Kappa8+Kappa9+kd))+Kappa10*(Kappa4*(Kappa6+kd)*(Kappa7+Kappa8+kd)+(Kappa5+Kappa6+kd)*(Kappa3*(Kappa8+kd)+kd*(Kappa7+Kappa8+kd))))))

[B_3_]_s_ = -((Kappa7*Kappa9*(Kappa4*(Kappa6+kd)+2*kd*(Kappa5+Kappa6+kd))*(f*Kappa1*(Kappa6+kd)*(Kappa10*((-Kappa3)*(Kappa8+kd)+kd*(Kappa7+Kappa8+kd))+kd*(Kappa7*(Kappa9+kd)-Kappa3*(Kappa8+Kappa9+kd)+kd*(Kappa8+Kappa9+kd)))+kd*(Kappa4*(Kappa6+kd)*(Kappa7*(Kappa9+kd)+kd*(Kappa8+Kappa9+kd))+kd*(Kappa5+Kappa6+kd)*(Kappa7*(Kappa9+kd)+Kappa3*(Kappa8+Kappa9+kd)+kd*(Kappa8+Kappa9+kd))+Kappa10*(Kappa4*(Kappa6+kd)*(Kappa7+Kappa8+kd)+(Kappa5+Kappa6+kd)*(Kappa3*(Kappa8+kd)+kd*(Kappa7+Kappa8+kd))))))/(Kappa2*(Kappa4*(Kappa6+kd)+2*kd*(Kappa5+Kappa6+kd))^2*(Kappa10*(Kappa8+kd)+kd*(Kappa8+Kappa9+kd))^2-Kappa1*(Kappa6+kd)*(Kappa10*((-Kappa3)*(Kappa8+kd)+kd*(Kappa7+Kappa8+kd))+kd*(Kappa7*(Kappa9+kd)-Kappa3*(Kappa8+Kappa9+kd)+kd*(Kappa8+Kappa9+kd)))*(Kappa4*(Kappa6+kd)*(Kappa7*(Kappa9+kd)+kd*(Kappa8+Kappa9+kd))+kd*(Kappa5+Kappa6+kd)*(Kappa7*(Kappa9+kd)+Kappa3*(Kappa8+Kappa9+kd)+kd*(Kappa8+Kappa9+kd))+Kappa10*(Kappa4*(Kappa6+kd)*(Kappa7+Kappa8+kd)+(Kappa5+Kappa6+kd)*(Kappa3*(Kappa8+kd)+kd*(Kappa7+Kappa8+kd))))))

[F]_s_ = (f*Kappa2*(Kappa4*(Kappa6+kd)+2*kd*(Kappa5+Kappa6+kd))^2*(Kappa10*(Kappa8+kd)+kd*(Kappa8+Kappa9+kd))^2+kd*(Kappa4*(Kappa6+kd)*(Kappa7*(Kappa9+kd)+kd*(Kappa8+Kappa9+kd))+kd*(Kappa5+Kappa6+kd)*(Kappa7*(Kappa9+kd)+Kappa3*(Kappa8+Kappa9+kd)+kd*(Kappa8+Kappa9+kd))+Kappa10*(Kappa4*(Kappa6+kd)*(Kappa7+Kappa8+kd)+(Kappa5+Kappa6+kd)*(Kappa3*(Kappa8+kd)+kd*(Kappa7+Kappa8+kd))))^2)/(Kappa2*(Kappa4*(Kappa6+kd)+2*kd*(Kappa5+Kappa6+kd))^2*(Kappa10*(Kappa8+kd)+kd*(Kappa8+Kappa9+kd))^2-Kappa1*(Kappa6+kd)*(Kappa10*((-Kappa3)*(Kappa8+kd)+kd*(Kappa7+Kappa8+kd))+kd*(Kappa7*(Kappa9+kd)-Kappa3*(Kappa8+Kappa9+kd)+kd*(Kappa8+Kappa9+kd)))*(Kappa4*(Kappa6+kd)*(Kappa7*(Kappa9+kd)+kd*(Kappa8+Kappa9+kd))+kd*(Kappa5+Kappa6+kd)*(Kappa7*(Kappa9+kd)+Kappa3*(Kappa8+Kappa9+kd)+kd*(Kappa8+Kappa9+kd))+Kappa10*(Kappa4*(Kappa6+kd)*(Kappa7+Kappa8+kd)+(Kappa5+Kappa6+kd)*(Kappa3*(Kappa8+kd)+kd*(Kappa7+Kappa8+kd)))))

The differential equations describing the changes in the concentrations of C*i*’s and F over time are

 (S24)

The steady-state solutions are:

Solution 1 (S25) (extinction):

[C_1_]_s_ = 0

[C_2_]_s_ = 0

[F]_s_ = f

Solution 2 (S26) (survival):

[C_1_]_s_ = -(((Kappa3-kd)*(f*Kappa1*(-Kappa3+kd)+kd*(Kappa3+Kappa4+kd)))/(Kappa1*(Kappa3-kd)*(Kappa3+Kappa4+kd)+Kappa2*(Kappa4+2*kd)^2))

[C_2_]_s_ = -(((Kappa4+2*kd)*(f*Kappa1*(-Kappa3+kd)+kd*(Kappa3+Kappa4+kd)))/(Kappa1*(Kappa3-kd)*(Kappa3+Kappa4+kd)+Kappa2*(Kappa4+2*kd)^2))

[F]_s_ = (kd*(Kappa3+Kappa4+kd)^2+f*Kappa2*(Kappa4+2*kd)^2)/(Kappa1*(Kappa3-kd)*(Kappa3+Kappa4+kd)+Kappa2*(Kappa4+2*kd)^2)

**Supplemental Table**

|  | | **Case ID** | | | |
| --- | --- | --- | --- | --- | --- |
|  |  | **(a)** | **(b)** | **(c)** | **(d)** |
| **Parameters** | ***k*_1_** (mol^–1^∙L∙unit time^–1^) | 2.00×10^–1^ | 2.00×10^–2^ | 1.00×10^21^ | 1.00×10^21^ |
|  | ***k*_2_** (mol^–1^∙L∙unit time^–1^) | 2.00×10^4^ | 2.00×10^–1^ | 1.00×10^23^ | 1.00×10^23^ |
|  | ***k*_3_** (unit time^–1^) | 9.90×10^–1^ | 9.50 | 9.00×10^–2^ | 9.90×10^–1^ |
|  | ***k*_4_** (unit time^–1^) | 1.00×10^–1^ | 1.00×10^–1^ | 3.00×10^–2^ | 3.00×10^–2^ |
|  | ***k*_d_** (unit time^–1^) | 1.00×10^–3^ | 1.00×10^–3^ | 2.00×10^–2^ | 2.00×10^–2^ |
|  | ***f*** (mol∙L^–1^) | 5.00 | 5.00 | 8.30×10^–22^ | 9.96×10^–22^ |
| ***U*(*k*_1_, *k*_2_, *k*_3_, *k*_4_, *k*_d_, *f*)** (mol∙L^–1^) | | 4.85×10^–4^ | –2.27×10^–1^ | 1.31×10^–23^ | 9.02×10^–23^ |
| **Steady-state concentration** (mol∙L^–1^) | **[A]_s_** | 4.95×10^–5^ | 2.35×10^–3^ | 7.53×10^–24^ | 4.80×10^–24^ |
|  | **[B]_s_** | 4.85×10^–4^ | 2.21×10^–1^ | 1.36×10^–23^ | 9.51×10^–23^ |
|  | **[A]_s_ + [B]_s_** | 5.35×10^–4^ | 2.23×10^–1^ | 2.11×10^–23^ | 9.99×10^–23^ |
|  | **[C]_s_** | 4.99×10^–5^ | 4.50×10^–1^ | 8.02×10^–24^ | 9.67×10^–24^ |
| **Steady-state molecule count in a 1 L CSTR** | ***n*_s,A_** | 2.98×10^19^ | 1.42×10^21^ | 4.54 ≈ 5 | 2.89 ≈ 3 |
|  | ***n*_s,B_** | 2.92×10^20^ | 1.33×10^23^ | 8.16 ≈ 8 | 57.3 ≈ 57 |
|  | ***n*_s,A_** **+ *n*_s,B_** | 3.22×10^20^ | 1.34×10^23^ | 12.70 ≈ 13 | 60.2 ≈ 60 |
|  | ***n*_s,C_** | 3.01×10^19^ | 2.71×10^23^ | 4.83 ≈ 5 | 5.82 ≈ 6 |
| **Effect of side reaction** | **Short-term** | Beneficial | Harmful | Beneficial | Harmful |
|  | **Long-term** |  |  |  | Beneficial |

**Table S1. Numerical examples of reversible side reactions in open reactors with different properties.** Four examples are shown, two (a, b) with high concentrations that could validly be modeled in a 1 L CSTR using mass action kinetics, and two (c, d) with low concentrations that result in low integer counts of molecules in a 1 L CSTR, requiring use of the Gillespie algorithm.
